# Measuring Paranoid Beliefs in Adolescents: A Comparison of the Revised-Green et al.’s Paranoid Thoughts Scale (R-GPTS) and the Bird Checklist of Adolescent Paranoia (B-CAP)

**DOI:** 10.1007/s10802-024-01187-9

**Published:** 2024-04-03

**Authors:** B. Schlier, L. Ellett, E. Thompson, B. Gaudiano, K. Krkovic, J. L. Kingston

**Affiliations:** 1 Clinical Child and Adolescent Psychology and Psychotherapy, Institute of Psychology, University of Wuppertal, Wuppertal, Germany; 2 School of Psychology, University of Southampton, Southampton, UK; 3 Department of Psychiatry & Human Behavior, Brown University and Butler Hospital, Providence, Rhode Island USA; 4 Department of Psychology, Royal Holloway, University of London, Surrey, UK

**Keywords:** Paranoid beliefs, Adolescents, R-GPTS, B-CAPS, Psychometric assessment

## Abstract

Research on paranoid beliefs in adolescents is in its infancy. Valid and reliable assessments are essential to advancing the field, yet there is no current consensus as to which are optimal to use in this population. This study compared the psychometric properties of two measures of paranoia in a general population adolescent sample. A cross-sectional study with quota sampling (gender and age) recruited adolescents (14–17 years) from the UK (n = 262) and USA (n = 200), who completed the Revised Green et al. Paranoid Thoughts Scale (R-GPTS) and the Bird Checklist for Adolescent Paranoia (B-CAP). We assessed factor structures, intercorrelations, overlap of participants identified as at-risk for paranoid thoughts via both scales, convergent validity (scales with one another) and discriminant validity (distress, wellbeing, bullying and discrimination). Both scales performed equally well in terms of factorial validity. Intercorrelations between the subscales and with general distress were high for both measures. However, a substantial percentage of participants were identified as having paranoid beliefs according to the R-GPTS *but not* the B-CAP. Furthermore, the B-CAP showed a very high correlations (0.69 ≤ r ≤ 0.79) with self-reported bullying experiences, which bordered on multicollinearity. Findings highlight the possibility that B-CAP may risk confounding paranoid beliefs with exposure to bullying more so than R-GPTS, and that B-CAP may miss instances of elevated paranoia that are captured by the R-GPTS. Future research needs to further explore this by validating both scales with an external (e.g., interview-based) criterion for paranoia.

**Supplementary Information:**

The online version contains supplementary material available at 10.1007/s10802-024-01187-9.

## Introduction

Paranoia describes unfounded beliefs that others intend to cause you harm (Freeman et al., 2021). Paranoia exists on a continuum, ranging from common concerns of suspicion and mistrust to persecutory delusions, which are more common in treatment seeking groups (Elahi et al., 2017). Paranoia has developmental routes in adolescence, with one in five reporting weekly paranoid beliefs, which are associated with reduced self-esteem and well-being over time (Kingston et al., 2022) as well as with suicidality (Bettes & Walker, 1986). Paranoid beliefs are more prevalent (≈ 33%) in treatment-seeking adolescents, characterized by increased psychological distress (Bird et al., 2021). Effective measurement of paranoia in adolescents is central to understanding its prevalence, nature and impact, as well as for identifying individuals who may require support. To date, however, there is no consensus as to which measures are most appropriate across different settings and age groups. Here, we focus on the use of self-report questionnaires to measure paranoia in adolescents. So far in the literature, this has included the use of adult questionnaires (e.g., 18+) either with no adaptations (e.g., Community Assessment of Psychotic Experiences, Stefanis et al., 2002; Paranoia Scale, Fenigstein & Vanable, 1992; Revised Green et al. Paranoid Thoughts Scale [R-GPTS], Freeman et al., 2021), or with minor changes to age-appropriate language (e.g., Specific Psychotic Experiences Questionnaire, Ronald et al., 2014). Additionally, a small number of questionnaires have been developed specifically for assessing paranoia in child and adolescent groups (e.g., Social Mistrust Scale, 8–14 year olds (Wong et al., 2014), Bird Checklist for Adolescent Paranoia; B-CAP (Bird et al., 2020) for 11–17 year olds).

The R-GPTS, formerly GPTS (Green et al., 2008), is currently considered to be the most valid and accurate paranoid beliefs questionnaire for use in general population and clinical adult samples (Statham et al., 2019). GPTS items were informed by the paranoia hierarchy, whereby rarer and more severe paranoia beliefs are thought to build on common social-evaluative concerns, with an ideas of reference subscale (milder social-evaluative concerns, Part A, e.g., “People definitely laughed at me behind my back“) and a persecutory ideation subscale (Part B, e.g., “People wanted me to feel threatened, so they stared at me“). The R-GPTS is a recent refinement based on factor analyzing aggregated datasets from general population and clinical samples. In adults, the R-GPTS has evidenced excellent psychometric properties (Statham et al., 2019) and -at least in part- validity and invariance across different cultures and language versions (Schlier et al., 2024). In adolescents (Bird et al., 2017; Korver-Nieberg et al., 2013) and mixed samples (e.g., of adolescents and adults from 12–34, Williams et al., 2023), the R-GPTS and GPTS have been used without adaptation, but there is limited information on reliability and validity in this group, with only GPTS persecution items being shown to be invariant across age groups 13–21 years (Freeman et al., 2021).

Researchers advocating the use of adolescent specific measures highlight the possibility of phenomenological differences in adolescent versus adult paranoid beliefs (Bird et al., 2020). For example, development of the B-CAP (Bird et al., 2020) yielded three subscales (social harm, physical harm and conspiracy ideas) assessing frequency of paranoid beliefs (*never* to *all of the time*). Items were generated based on the clinical expertise of the researchers, previous paranoia measures, and consultation with young people. Some items contextualize paranoid beliefs within the school setting (e.g., “people are trying to embarrass me in class on purpose”), others give reference to social media (e.g., “I am sure people are gossiping about me on social media”), and others to friendship groups (e.g., “my friends or partner are ignoring my messages to upset me on purpose”). These items are interspersed with more generic items (e.g., “I think people are lying to me on purpose”). The B-CAP has been validated in two UK adolescent samples (mixed community and treatment seeking participants; Bird et al, 2021, 2020), with Item Response Analysis suggesting that items are discriminative of paranoia across the spectrum of experience. However, only a small (n = 7) number of participants had suspected psychosis (Bird et al., 2020).

The psychometric assessment of R-GPTS and B-CAP in adolescents thus remains somewhat in its infancy. This study thus aimed to compare these two measures in a general population adolescent (14–17 years) sample, using data from two high-income countries (UK and USA). We chose the R-GPTS as the most well-established measure of paranoid beliefs in adults, which has been extended to adolescent groups, and the B-CAP as the most thoroughly validated paranoia questionnaire for adolescents. Furthermore, and importantly, both questionnaires aim to measure paranoia across the severity continuum, making them comparable in this regard. The specific focus of the comparison was to: (1) test the validity of their factor structure using confirmatory factor analysis (CFA); (2a) examine the intercorrelation of the R-GPTS and B-CAP and (2b) the overlap of participants identified as at-risk for paranoid thoughts via R-GPTS and B-CAP cutoffs to determine whether both scales (and their subscales) tap into the same construct; and (3) assess both scales for convergent and discriminant validity. While intercorrelation between the paranoia measures, as well as intercorrelations within their respective subscales, served as indicators of convergent validity, discriminant validity was evaluated by examining correlations with global measures of mental health (i.e., distress, wellbeing) and with self-reports of bullying and discrimination. We chose to include these latter constructs because they are considered to be distinct from paranoia (i.e., bullying and discrimination are recounts of hostile external experiences whereas paranoia centers on the internal appraisal), but also show considerable content overlap (i.e., quantifying acts of hostility intentionally aimed at one person). Thus, we reasoned that a measure of paranoid beliefs must be sufficiently distinguishable from accounts of actual threat and victimization.

## Methods

### Participants

UK (n = 262) and USA (n = 200) adolescents (14–17 years) were recruited through Qualtrics, an online participant recruitment service, with quota sampling to ensure a 50:50 gender and age (14–15 and 16–17) split.

### Materials

*Descriptive and socio-demographic variables* included age, gender, household income, country of birth and self-reported current diagnosis of a mental health disorder (yes/no).

The *R-GTPS* (Freeman et al., 2021) measures ideas of reference (8 items) and of persecution (10 items). Items, rated on a 5-point scale (*0–not at all* to *4–totally*), exhibit reliability across the paranoia continuum. Subscale sum-scores were used. R-GPTS authors reported excellent internal consistency (α = 0.90–0.95, Freeman et al., 2021) and its two-factor structure has been recently replicated in a mixed sample of adolescents and adults at risk for psychosis (Williams et al., 2023). Freeman and colleagues (2021) advised on the following categorizations based on latent trait analysis: Persecution subscale (range 0–40): average 0–5, elevated 6–10, moderately severe 11–17, severe 18–27, very severe 28+; Ideas of reference subscale (0–32) average 0–9, elevated 10–15, moderately severe 16–20, severe 21–24, very severe 25+. Of importance, the rationale behind at least some of these category threshold is linked to validated clinical cutoffs: Freeman et al. (2021) describe > 11 in persecutory delusions (moderately severe) as overall optimal cut-off to discriminate patients with persecutory delusions from non-clinical participants, but advise using ≥ 18 in persecutory delusions (severe) as more accurate cut-off, since it minimizes false positive classification of clinical levels of persecutory delusions, while still correctly identifying most patients. There is no corresponding cut-off for ideas of reference.

The *B-CAP* (Bird et al., 2020) measures the frequency of paranoid thinking in adolescents via three factors: social harm (8 items, score range: 0–40), conspiracy (5 items, score range: 0–25), and physical threat (5 items, score range: 0–25) and using a scale ranging from 0-*never* to 5-*all of the time*. We used the sum-scores of the three factors as well as the grand total score (score range; 0–90) with higher scores indicating greater paranoia. Authors reported excellent internal consistency (Total: α = 0.92) and the following categories for total scores (range 0–90): average 0–22, mildly elevated 23–39, moderate 40–53, high 54–70, severe 71–90. As yet, there are no published guidelines for categorizing subscale scores and no cut-offs (i.e., for clinical levels of  persecutory delusions) are available.

*The Depression Anxiety Stress-short form* (DASS-21, Henry & Crawford, 2005) measures depression, anxiety and tension/stress (7 items per subscale) over the previous week using a scale from 0-*Did not apply to me at all* to 3-*Applied to me very much/most of the time*. Authors reported excellent internal consistency (α = 0.93; Henry & Crawford, 2005). The total score (range: 0–63) as well as subscale scores for depression, anxiety, and stress (range: 0–21, respectively) were calculated. The DASS-21 has been validated in general population adolescents across various cultural contexts, demonstrating good convergent validity (Evans et al., 2020; Evans et al., 2021), factorial invariance (Mellor et al., 2015), internal reliability (α > 0.80) and supporting the use of the composite distress score (Jovanović, et al., 2021; Patrick et al., 2010).

The *Warwick-Edinburgh Mental Wellbeing Scale* (WEMWBS; Tennant et al., 2007) measures general population wellbeing using 14 item that are rated on a 5-point scale ranging from 1-*none of the time* to 5-*all of the time*. Reliability (α = .87) and good criterion and factorial validity has been demonstrated in a large sample of adolescents (Clarke et al., 2011). Total scores (range: 14–70) were used.

The *Brief Self-Report Measure of Adolescent Bullying* (Murray et al., 2021) measures bullying victimization in the last 12-months. Participants are presented five increasingly sever examples of being bullied (e.g., purposefully ignored; hit, bitten, kicked) and asked to estimate how many times over the last year this has happened to them (0-*never*, 1–*1 to 2 times*, 2–*3 to 10 times*, 4-*about once a month*, 5-*about once a week*, 5*-(almost) every day*. Authors reported acceptable internal consistency in their original validation sample (Omega: 0.52–0.77; Murray et al., 2021) for the total score used in this study (score range: 0–25).

The *Everyday Discrimination Scale* (EDS; Willaims et al., 1997) assesses discriminatory experiences without specific reference to any particular domain, thus capturing discrimination across a variety of areas. Five-items assess the frequency (6-point scale from 0-*never* to 5-*almost everyday*) of discriminatory experiences, ranging from mild (treated with less courtesy) to more chronic experiences (threatened or harassed). Scores range from 6–30 with higher scores indicating higher discrimination. Good internal consistency has been reported (α = 0.88; Kim et al., 2014).

### Procedure

Ethical approval was obtained from the host sites (Butler Hospital and Royal Holloway, University of London). Adults pre-registered with research recruitment panels and who reported living with their adolescent child were contacted, via Qualtrics, to seek permission to invite their adolescent child to take part in the survey. With parental consent, the adolescent child was invited to read the information sheet and consent form. Consenting adolescents then completed initial screener items to check eligibility (i.e., based on quotas). Eligible participants were then given access to the online questionnaires. Participants were reimbursed for their time. To help prevent missing data, participants were required to respond to all questions. To enhance data accuracy, participants had to correctly respond two attention checks that were distributed through the survey. Completion time was also monitored and those taking less than half the median completion time were excluded by Qualtrics at source. Participants with a geographical location that did not correspond with the stated location, and/or who did not consent to their data being used, and/or dropped out without completing all measures were excluded at source by Qualtrics.

### Data Analysis

Factorial validation was performed with CFA by testing for the goodness-of-fit to a predefined two factor structure for the R-GPTS and three factor structure for the B-CAP. Each of these models was compared to a more conservative, one factor model. We used the fit indices and established cut-off criteria (Hu & Bentler, 1999) for CFI (good fit: CFI > 0.95, sufficient fit: CFI > 0.90), RMSEA (good fit: RMSEA < 0.06), and SRMR (good fit: SRMR < 0.08). To account for non-normal distribution, all CFA were calculated with maximum likelihood estimation with robust standard errors and a Satorra-Bentler scaled test statistic.

Next, to assess overlap between the two scales, we first calculated correlations between R-GPTS and B-CAP subscale scores. Additionally, we explored whether participants identified as likely to have paranoid beliefs according to one scale would also be identified as such by the other. To this end, we first used the predefined cut-off for likely presence of persecutory delusions (R-GPTS persecutory delusion subscale sum-score ≥ 18, corresponding to 1.10 SD above the average score [“severe”] in the latent trait of persecutory beliefs, Freeman et al., 2021) to group participants into people with and without persecutory delusions according to R-GPTS. Since no singular diagnostic cut-off for likely presence of persecutory delusions exists for the B-CAP, we used the cut-off for the category “moderate levels” of paranoia (i.e., ≥ 40). We chose this as a reasonable comparison because a B-CAP total sum-score ≥ 40 corresponds to 1.45 SD above the average in latent trait paranoia for adolescents, with the next lower category having a pre-defined threshold corresponding to 0.75 SD above the latent trait average (Bird et al., 2020). This cut-off was thus used to split the sample into two groups in a similar way to the R-GPTS. Using this categorization, we tested whether there was a significant percentage of participants identified as likely to be experiencing paranoid beliefs by one scale, but not the other. For R-GPTS ideas of reference subscale, we used the previously established 1.10 SD above average to mirror the cutoff used for R-GPTS ideas of persecution. Moreover, to double check that any divergences between the two scales are not merely an artifact of the 0.35 SD difference in the definition of the cut-offs, we repeated the comparison with cut-off variations more similar in their population-based definition, namely (1) the R-GPTS moderate cut-offs (SD ≥ 0.8 above average) and B-CAP total slightly elevated (SD ≥ 0.75 above average) as well as (2) the R-GPTS very severe cut-offs vs B-CAP moderate cutoff (both SD ≥ 1.45 above average).

Finally, for convergent and discriminant validity, we calculated correlations between all R-GPTS/B-CAP subscales (convergent validity) and DASS-21 total/subscale scores, the WEMWBS, bullying and discrimination experience scores (discriminant validity).

## Results

Analysis of the item scores yielded one participant with a pattern of extreme answer (all items of a given scale either at the upper or lower end of the scoring), who was excluded from the analysis (final sample n = 461). Sample characteristics are summarized in Table 1.

**Table 1:** Demographic data of the analyzed sample (n = 461)

Variable	n	%	M	SD
Gender				
Female	229	49.7%		
Male	229	49.7%		
Trans female	1	0.2%		
Trans male	1	0.2%		
Other	1	0.2%		
Age			15.51	1.13
Ethnicity				
White	369	80.0%		
Black	31	6.7%		
Hispanic/Latino	31	6.7%		
Asian	14	3.0%		
Mixed	12	2.6%		
Other	4	0.9%		
SES				
Below average	116	25.2%		
Average	263	57.0%		
Above average	50	10.8%		
Don’t know	32	6.9%		
Mental health diagnosis	90	19.5%		
Currently taking psychiatric medication	48	10.4%		

Mean paranoia scores were within the average category for both R-GPTS (ideas of reference: M = 7.14, SD = 8.63; persecutory delusions: M = 6.29, SD = 9.52) and B-CAP-scores (B-CAP-total: M = 8.92, SD = 14.55), however, distribution of B-CAP (skew = 2.37) scores were more skewed when compared to R-GPTS scores (ideas of reference: skew = 1.14; persecutory delusions: skew = 1.60), with the subscales B-CAP conspiracy (skew = 3.25) and B-CAP physical harm (skew = 2.63) showing the most deviation from normal distribution (an extended overview of descriptive statistics for all measures and scale reliability in this sample are added as a supplement to this article, see Table S1).

### Factor Validity

CFA of R-GPTS and B-CAP showed sufficient fit according to CFI and SRMR, but not RMSEA for both the two-factor model of the R-GPTS and the three-factor model of the B-CAP (Table 2). Furthermore, all items loaded sufficiently high on their respective factor (> 0.600) and no individual item stood out in either scale as particularly poor (For CFA-loadings and a complementary EFA, see supplemental material). The fit of the two-factor model of the R-GPTS and the three-factor model of the B-CAP were significantly better than their one-factor model solution (BIC/AIC reduction > 10). In the online supplement, item characteristics (mean, standard deviation, and distribution of answers) are summarized for both the R-GPTS (Table S2) and the B-CAP (Table S3).

**Table 2:** Results of the confirmatory factor analysis (CFA) of the R-GPTS and the B-CAP

Factor Structure	Robust CFI (> 0.90)	Robust RMSEA (< 0.06)	RMSEA 90%-CI	SRMR (< 0.08)	AIC	BIC	loadings
R-GPTS							
1 Factor	0.859	0.139	0.128–0.151	0.058	18911.72	19060.52	0.708–0.860
2 Factors	0.906	0.114	0.103–0.126	0.051	18493.26	18646.19	0.733–0.879
B-CAP							
1 Factor	0.809	0.143	0.135–0.161	0.07	17819.92	17968.73	0.667–0.840
3 Factors	0.933	0.089	0.074–0.103	0.064	16937.02	17098.22	0.647–0.916

### Overlap of R-GPTS and B-CAP (Convergent Validity)

Correlations between subscales within one measure (R-GPTS or B-CAP) were very large (R-GPTS: r = 0.84; B-CAP: 0.70 ≤ r ≤ 0.80). Correlations between subscales of different paranoia measures were at the same level for B-CAP social harm (0.73 ≤ r ≤ 0.75), but a little lower for B-CAP conspiracy beliefs (0.58 ≤ r ≤ 0.68) and B-CAP physical threat (0.55 ≤ r ≤ 0.60; see Table 3).

**Table 3:** Correlation between R-GPTS and B-CAP as well as between both scores and discriminant validity parameters.

	RGPTS Ideas of reference	RGPTS Persecutory beliefs	B-CAP Social Harm	B-CAP Conspiracy beliefs	B-CAP Physical Threat
Convergent validity					
RGPTS Persecutory beliefs	0.85				
B-CAP Social Harm	0.73	0.75			
B-CAP Conspiracy beliefs	0.58	0.68	0.80		
B-CAP Physical Threat	0.55	0.60	0.70	0.75	
Discriminant validity					
DASS Total	0.62	0.65	0.73	0.64	0.70
DASS Depression	0.59	0.59	0.69	0.59	0.62
DASS Anxiety	0.60	0.65	0.71	0.66	0.73
DASS Stress	0.58	0.61	0.67	0.57	0.65
WEMWBS Wellbeing	-0.23	-0.25	-0.26	-0.10	-0.18
Bullying	0.59	0.66	0.79	0.79	0.69
Discrimination experiences	0.40	0.40	0.41	0.44	0.40

All correlations were calculated based on the full sample (n = 461) and all correlations were statistically significant significant with all *p* < 0.001, except for the association between B-CAP conspiracy beliefs and WEMWBS Wellbeing, *p* = 0.030)

**Fig. 1:**
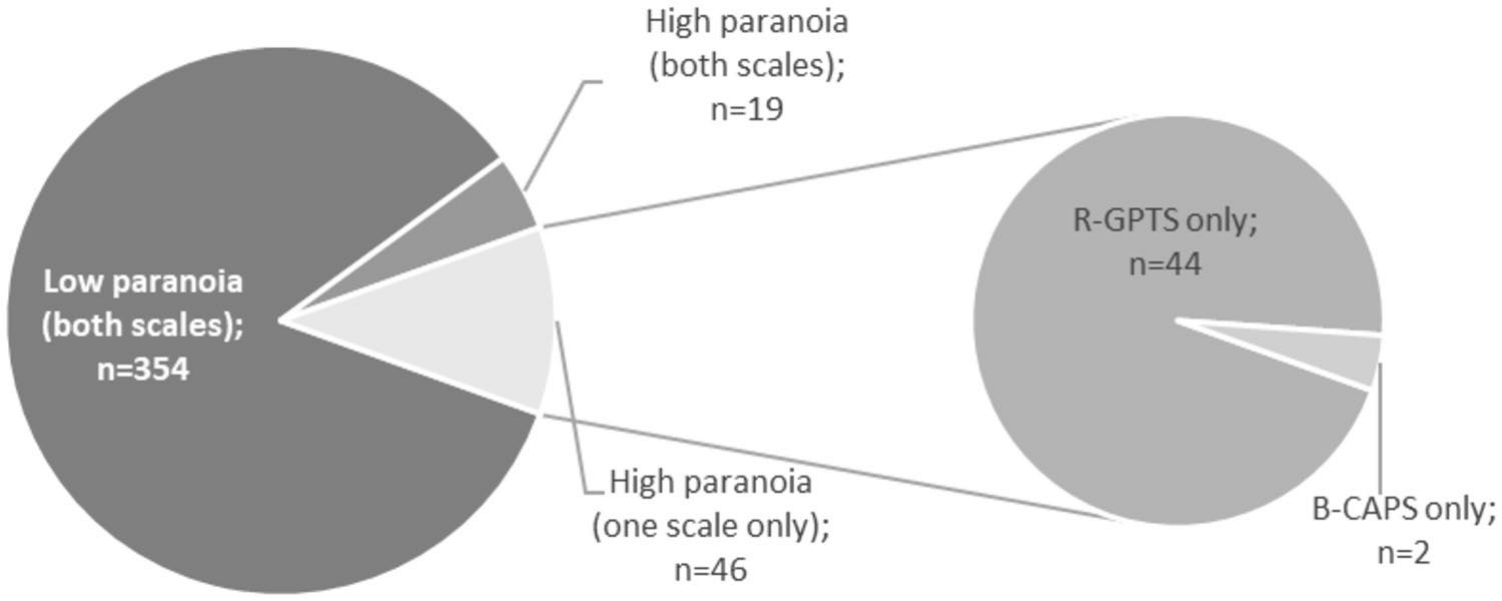
Percentage of participants below and above cut-offs (R-GPTS persecutory delusions and B-CAP total)

Categorization according to cut-offs (Table 4) yielded 63 participants (13.6%) with high levels of paranoia according to R-GPTS. Using the B-CAP cut-off, only 21 participants (4.6%) with high levels of paranoia were identified. A comparison of both categorizations (see Fig. 1) showed that most participants were identified as below clinical levels of paranoid beliefs according to both scales’ cut-off (n = 354; 76.8%, 95%-CI: 72.7–80.6%). Of those identified as having high/clinical levels of paranoia, only a small number were identified in both scales (n = 19; 4.1%, 95%-CI: 2.5–6.4%), whereas most participants (n = 44, 9.5%, 95%-CI: 7.0-12.6%) showed high levels according to the R-GPTS only, and almost no one showed high levels according to the B-CAP only (n = 2, 0.4%, 95%-CI: 0.1–1.6%). Stability analyses with variations in the B-CAP total and R-GPTS persecutory threat group cut-offs yielded comparable patterns of results as the corresponding main analysis, with high levels for B-CAP only remaining the lesser frequent category than the high levels for R-GPTS only in all variations (see Table 4).

**Table 4:** Overlap between high vs. low paranoia groups based on R-GPTS and B-CAP cutoffs

R-GPTS scale	B-CAP scale	Both low	B-CAP high	RGPTS high	Both high	% overlap high & low scorers categorization	% overlap high scorers only
Main Analyses							
Persecution^a^	Total score^b^	396 (85.9%)	2 (0.4%)	44 (9.5%)	19 (4.1%)	90.02%	29.23%
Ideas of reference^c^	Total score^b^	402 (87.2%)	4 (0.9%)	38 (8.2%)	17 (3.7%)	90.89%	28.81%
Stability analyses lower cut-offs^d^							
Persecution	Total score	339 (73.5%)	15 (3.3%)	54 (11.7%)	53 (11.5%)	85.03%	43.44%
Ideas of reference	Total score	356 (77.2%)	17 (3.7%)	37 (8.0%)	51 (11.0%)	88.29%	48.57%
Stability analyses higher cut-offs^e^							
Persecution	Total score	423 (91.8%)	7 (1.5%)	17 (3.7%)	14 (3.0%)	94.79%	36.84%
Ideas of reference	Total score	422 (91.5%)	12 (2.6%)	38 (8.2%)	12 (2.6%)	94.14%	19.35%

^a^the cut-off for R-GPTS persecution was the predefined cut-off for likely presence of persecutory delusions (1.10 SD above latent trait average of the validation sample)

^b^the cut-off for B-CAP total was based on predefined group-cut offs from the validation sample, using the cut-off for the category “moderate levels” of paranoia (corresponding to 1.45 SD above latent trait average for adolescents) to match the R-GPTS cutoff (≥ 18, corresponding to 1.10 SD above latent trait average of the validation sample) as closely as possible

^c^the cut-off for R-GPTS ideas of reference was defined analogous to the Persecution subscale (1.10 SD above latent trait average of the validation sample)

^d^lower cut-offs for both scales were chosen with the moderate-category threshold for R-GPTS (0.80 SD above latent trait average of the validation sample, coinciding with the secondary, optimal accuracy cut-off for persecutory delusions as established by Freeman et al., 2021) and the mildly elevated category for B-CAPS (0.75 SD above latent trait average of the validation sample)

^e^higher cut-offs correspond to 1.45 SD above latent trait average in the corresponding validation samples for both R-GPTS (“very severe” category) and B-CAP (“moderate” category)

### Discriminant Validity

Regarding discriminant validity (see Table 3), correlations were notably lower than the convergent validity scores for Wellbeing (-0.25 ≤ r≤-10) and, to a lesser degree, for discrimination experiences (0.40 ≤ r ≤ 0.44). Large correlations with DASS-scores and Bullying were found for both scales, with R-GPTS showing marginally lower values (correlations with DASS-scores: 0.58 ≤ 0.65; correlations with bullying; 0.59 ≤ r ≤ 0.65) than the B-CAP (correlations with DASS-scores: 0.57 ≤ 0.73; correlations with bullying; 0.69 ≤ r ≤ 0.79). For the R-GPTS, correlations with DASS-scores and bullying were roughly in the same range as correlations between R-GPTS and B-CAP scores, but still below correlations within R-GPTS-subscales. For the B-CAP, by contrast, correlations with DASS-scores and bullying exceeded the correlation with the R-GPTS subscales and were often within the same range as intercorrelations of B-CAP-subscales.

## Discussion

This study compared the psychometric properties of R-GPTS and the B-CAP in a UK and USA general adolescent population. Both scales exhibited factor structures that were consistent with their original validation papers. Furthermore, intercorrelations between the subscales of both measures were high. However, both scales also showed high correlations with general levels of distress and bullying, thus indicating generally low levels of discriminant validity. High correlations with these discriminant validation variables have previously been reported, although the values seem to constitute the upper boundary of prior findings: For example, recent systematic reviews have reported correlations between paranoia and childhood experiences of bullying that ranged from 0.19 to 0.59 in adolescent and adult samples (Jack & Egan, 2017) and correlations ranging from 0.16 to 0.61 for paranoia and discrimination in adult samples (Pearce et al., 2019). In the current paper, the problem of low discriminant validity was more pronounced in the B-CAP, where correlations with bullying were within the same range as the intercorrelation between the B-CAP subscales and above correlations of R-GPTS and B-CAP, highlighting a potential confounding effect in the B-CAP.

Further exploration of the overlap between both scales revealed a substantial percentage of the sample was identified as having paranoid beliefs according to the R-GPTS but not the B-CAP. When excluding participants with low scores on both scales, overlap dropped to 30–50% of participants scoring high on both measures. However, it is important to raise that in the absence of an established and validated B-CAP cut-off, we needed to choose one ourselves. In doing so, we purposefully selected a cut-off that corresponded as closely as possible to the cut-off used for R-GPTS; that is, 1.10 SD above the average for R-GPTS (validated by Freeman et al., 2021) versus 1.45 SD above the average for B-CAP (based on the expected scores derived from a graded response model by Bird et al., 2020), which corresponded to group descriptors of ‘severe’ for R-GPTS and ‘moderate’ for the B-CAP. Of importance, the overall pattern of results remained consistent, when lower or higher variation of cut-offs were chosen. In sum, we aimed to achieve a roughly similar cut-off based on latent variable SDs, but it needs to be taken into account that the R-GPTS cut-off has been cross-validated with an external criterion (i.e., differentiating between participants with vs without confirmed persecutory delusions), whilst the B-CAP group descriptors are solely based on the distribution of latent trait and expected values.

A likely explanation for the discrepancy in identifying people with high paranoia is the difference in skew between R-GPTS and B-CAP. The less skewed distribution of the R-GPTS includes more people with higher scores relative to the B-CAP, resulting in more people identified as highly paranoid. The exact reason for this might be rooted in the item content of the to scales: The R-GPTS comprises beliefs about non-specified situations that range from mild suspiciousness (e.g., “I spent time thinking about friends gossiping about me”) to more severe persecutory beliefs (e.g., “I was sure someone wanted to hurt me”). Importantly, all items can be applied to a range of people or groups that the respondent might be thinking of. In contrast, some of the B-CAP items narrow the context to a situation involving a specific group of peers (e.g., “people are trying to embarrass me in class on purpose”). Whilst this representation of age-specific situations could make the B-CAP a more accessible measure, this could result in an oversight of paranoid beliefs rooted in other social interactions or paranoid beliefs held in absence of an average social life due to, for example, social withdrawal or avoidance. In selecting the most sensitive and appropriate measure, it may therefore be useful to consider the R-GPTS for assessing the wide spectrum of experience and possible risk for persecutory delusions, whereas the B-CAP may assess paranoid beliefs in a manner that is relatively more embedded within adolescent contexts (e.g., peers, school, social media). Considering the high correlation between B-CAP and bullying, researchers and clinicians may also need to consider the issue of discriminating between valid perceptions of harmful intent based on current victimization versus beliefs that have become exaggerated and generalized (i.e., paranoid beliefs). This is an issue that cuts across the scales, but that seems more problematic for the B-CAP. It may therefore seem prudent to select the R-GPTS over the B-CAP when studying the effect of bullying, social exclusion, or risk factors associated with social adversity on paranoid beliefs in adolescents. This way, autocorrelation between measures of social adversity and adolescent paranoia, and the risk of overestimating the influence of these putative risk factors, is reduced. Alternatively, it may be advisable to consider the use of interviews (e.g., Structured Interview of Psychosis-Risk Syndromes (SIPS), 12–17 years; Miller et al., 1999; Woods et al., 2014) to help tease apart adaptive beliefs founded in current adversity (i.e., bullying, discrimination) from paranoid beliefs.

### Limitations

Due to the cross-sectional nature of this study, it is not possible to ascertain whether the two scales measure paranoid beliefs that persist when potentially confounding social factors (e.g., bullying) decline or cease to occur. Future research would benefit from further assessment of the validity of these scales in adolescents. Important next steps include assessing the construct validity of the measures by comparing them to a structured clinical interview (e.g., SIPS; Miller et al., 1999) and/or to other more objective methods of assessment, such as paranoid interpretations towards neutrally programmed VR avatars, attributions of intentional harm assessed via ambiguous social scenarios (Calleja & Rapee, 2020) or by using in vivo social experiments. Additionally, by virtue of using a general population sample, low correlations between the rarer experiences (e.g., conspiracy beliefs, physical threat, persecution) might be the result of restricted variation in the sample. Furthermore, whereas clinical cut-offs for the R-GPTS have been pre-established (in adult samples) all other “clinical cut-offs” (R-GPTS reference, B-CAP) used in this study have been defined within this evaluation and based on the relative point of R-GPTS persecution cut-off in the distribution of latent trait scores. The definition of these cut-offs is thus somewhat arbitrary and a validated cut-off is needed to further investigate the clinical cut-off overlap. In order to determine clinical cut-offs for adolescents, future research with clinical samples is required. Finally, the generalizability of the findings is limited to predominantly White individuals living in high-income countries, as well as to young people whose parents were are registered with survey recruitment panels. Adults registered to earn money through recruitment panels may, for example, be more likely to be unemployed and/or to struggle financially. Thus, whilst the sample is representative on the basis of some sociodemographic characteristics (e.g., age and gender), other characteristics may limit generalizability. Future research would benefit from assessing the generalizability of these findings using large general population adolescent samples.

## Conclusion

This study offers the first comprehensive comparison of self-report measures of paranoid beliefs in adolescence. While both show promise in several regards (internal consistency, factor structure), our findings highlight some concerns regarding the B-CAP, namely, substantial construct overlap with bullying. Further validation is required. Future studies need to continue to investigate the validity of self-report measures for adolescent paranoia in more diverse samples including at risk/early psychosis patients and healthy controls and using repeated assessments to fully elucidate the validity of these scales as screening tool indicators of symptom severity in adolescents. Most importantly, future research needs to include an external criterion for validation of cut-offs and proximity to clinical status (in adolescents) for both scales in order to explore whether R-GPTS is over-identifying cases of elevated paranoid beliefs, or whether B-CAP might be under-identifying.

Based on our initial findings, we recommend that any decisions between R-GPTS and B-CAP for measuring paranoid beliefs in general population adolescents should be made in light of the specific aim and purpose of a planned assessment. For example, since our findings highlight the possibility that B-CAP may risk confounding paranoid beliefs with exposure to social adversity (e.g., bullying) more so than R-GPTS, the R-GPTS might be the assessment of choice in research investigating the association between social adversities and paranoia in order to avoid positively biased, inflated results. Finally, as the research in adolescent paranoia grows, other important considerations will include assessing dimensionality, such as conviction, distress and preoccupation (Woods et al., 2014), enhancing efforts to consult and collaborate with young people in determining how best to capture paranoid concerns during this developmental period and the use of more objective, yet ecologically valid assessments, to assist in further development and validation of self-report measures.

### Supplementary Information

Below is the link to the electronic supplementary material

Supplementary Material 1

## References

[CR1] Bettes, B. A., & Walker, E. (1986). Symptoms associated with suicidal behavior in childhood and adolescence. *Journal of Abnormal Child Psychology,**14*(4), 591–604. 10.1007/BF01260526 3782629 10.1007/BF01260526

[CR2] Bird, J. C., Fergusson, E. C., Kirkham, M., Shearn, C., Teale, A. L., Carr, L., & Freeman, D. (2021). Paranoia in patients attending child and adolescent mental health services. *Australian & New Zealand Journal of Psychiatry,**55*(12), 1166–1177. 10.1177/0004867420981416 33423520 10.1177/0004867420981416 PMC8649424

[CR3] Bird, J. C., Loe, B. S., Kirkham, M., Fergusson, E. C., Shearn, C., Stratford, H., & Freeman, D. (2020). The assessment of paranoia in young people: Item and test properties of the Bird Checklist of Adolescent Paranoia. *Schizophrenia Research,**220*, 116–122. 10.1016/j.schres.2020.03.046 32247744 10.1016/j.schres.2020.03.046

[CR4] Bird, J. C., Waite, F., Rowsell, E., Fergusson, E. C., & Freeman, D. (2017). Cognitive, affective, and social factors maintaining paranoia in adolescents with mental health problems: A longitudinal study. *Psychiatry research,**257*, 34–39. 10.1016/j.psychres.2017.07.023 28715666 10.1016/j.psychres.2017.07.023

[CR5] Calleja, R. L., & Rapee, R. M. (2020). Social threat sensitivity and its relationships with peer victimisation and internalising symptoms among adolescent girls. *Behaviour Research and Therapy,**133*, 103710. 10.1016/j.brat.2020.103710 32836111 10.1016/j.brat.2020.103710

[CR6] Clarke, A., Friede, T., Putz, R., Ashdown, J., Martin, S., Blake, A., & Stewart-Brown, S. (2011). Warwick-Edinburgh Mental Well-being Scale (WEMWBS): validated for teenage school students in England and Scotland. A mixed methods assessment. *BMC Public Health,**11*, 1–9. 10.1186/1471-2458-11-487 21693055 10.1186/1471-2458-11-487 PMC3141456

[CR7] Elahi, A., Algorta, G. P., Varese, F., McIntyre, J. C., & Bentall, R. P. (2017). Do paranoid delusions exist on a continuum with subclinical paranoia? A multi-method taxometric study. *Schizophrenia Research,**190*, 77–81. 10.1016/j.schres.2017.03.022 28318838 10.1016/j.schres.2017.03.022

[CR8] Evans, L., Haeberlein, K., Chang, A., & Handal, P. (2020). An evaluation of the convergent validity of and preliminary cutoff scores for the DASS-21 Total score as a measure of distress in adolescents. *Current Psychology,**41*, 4283–4290. 10.1007/s12144-020-00937-4 10.1007/s12144-020-00937-4

[CR9] Evans, L., Haeberlein, K., Chang, A., & Handal, P. (2021). Convergent Validity and Preliminary Cut-Off Scores for the Anxiety and Depression Subscales of the DASS-21 in US Adolescents. *Child Psychiatry & Human Development,**52*, 579–585. 10.1007/s10578-020-01050-0 32816139 10.1007/s10578-020-01050-0

[CR10] Fenigstein, A., & Vanable, P. A. (1992). Paranoia and self-consciousness. *Journal of personality and social psychology,**62*(1), 129. 10.1037/0022-3514.62.1.129 1538311 10.1037/0022-3514.62.1.129

[CR11] Freeman, D., Loe, B. S., Kingdon, D., Startup, H., Molodynski, A., Rosebrock, L., & Bird, J. C. (2021). The revised Green et al., Paranoid Thoughts Scale (R-GPTS): psychometric properties, severity ranges, and clinical cut-offs. *Psychological Medicine,**51*(2), 244–253. 10.1017/s0033291719003155 31744588 10.1017/s0033291719003155 PMC7893506

[CR12] Green, C. E. L., Freeman, D., Kuipers, E., Bebbington, P., Fowler, D., Dunn, G., & Garety, P. A. (2008). Measuring ideas of persecution and social reference: the Green et al. Paranoid Thought Scales (GPTS). *Psychological Medicine,**38*(1), 101–111. 10.1017/s0033291707001638 17903336 10.1017/s0033291707001638

[CR13] Henry, J. D., & Crawford, J. R. (2005). The short-form version of the Depression Anxiety Stress Scales (DASS‐21): Construct validity and normative data in a large non‐clinical sample. *British Journal of Clinical Psychology,**44*(2), 227–239. 10.1348/014466505x29657 16004657 10.1348/014466505x29657

[CR14] Hu, L. T., & Bentler, P. M. (1999). Cutoff criteria for fit indexes in covariance structure analysis: Conventional criteria versus new alternatives. *Structural Equation Modeling: A Multidisciplinary Journal,**6*(1), 1–55. 10.1080/10705519909540118 10.1080/10705519909540118

[CR15] Jack, A. H., & Egan, V. (2017). Trouble at school: a systematic review to explore the association between childhood bullying and paranoid thinking. *Psychosis,**9*(3), 260–270. 10.1080/17522439.2017.1340503 10.1080/17522439.2017.1340503

[CR16] Jovanović, V., Gavrilov-Jerković, V., & Lazić, M. (2021). Can adolescents differentiate between depression, anxiety and stress? Testing competing models of the Depression Anxiety Stress Scales (DASS-21). *Current Psychology,**40*(12), 6045–6056. 10.1007/s12144-019-00540-2

[CR17] Kim, G., Sellbom, M., & Ford, K. L. (2014). Race/ethnicity and measurement equivalence of the Everyday Discrimination Scale. *Psychological Assessment,**26*(3), 892. 10.1037/a0036431 24708076 10.1037/a0036431 PMC4152383

[CR18] Kingston, J. L., Parker, A., & Schlier, B. (2022). Effects of paranoia on well-being in adolescents: A longitudinal mediational analysis. *Schizophrenia Research,**243*, 178–180. 10.1016/j.schres.2022.03.009 35381516 10.1016/j.schres.2022.03.009

[CR19] Korver-Nieberg, N., Fett, A. K. J., Meijer, C. J., Koeter, M. W., Shergill, S. S., de Haan, L., & Krabbendam, L. (2013). Theory of mind, insecure attachment and paranoia in adolescents with early psychosis and healthy controls. *Australian & New Zealand Journal of Psychiatry,**47*(8), 737–745. 10.1177/0004867413484370 23553238 10.1177/0004867413484370

[CR20] Mellor, D., Vinet, E. V., Xu, X., Mamat, N. H. B., Richardson, B., & Román, F. (2015). Factorial invariance of the DASS-21 among adolescents in four countries. *European Journal of Psychological Assessment*. 10.1027/1015-5759/a000218 10.1027/1015-5759/a000218

[CR21] Miller, T. J., McGlashan, T. H., Woods, S. W., Stein, K., Driesen, N., Corcoran, C. M., & Davidson, L. (1999). Symptom assessment in schizophrenic prodromal states. *Psychiatric Quarterly,**70*, 273–287. 10.1023/a:1022034115078 10587984 10.1023/a:1022034115078

[CR22] Murray, A. L., Eisner, M., Ribeaud, D., Kaiser, D., McKenzie, K., & Murray, G. (2021). Validation of a brief self-report measure of adolescent bullying perpetration and victimization. *Assessment,**28*(1), 128–140. 10.1177/1073191119858406 31280595 10.1177/1073191119858406

[CR23] Patrick, J., Dyck, M., & Bramston, P. (2010). Depression Anxiety Stress Scale: is it valid for children and adolescents? *Journal of clinical psychology,**66*(9), 996–1007. 20694962 10.1002/jclp.20696

[CR24] Pearce, J., Rafiq, S., Simpson, J., & Varese, F. (2019). Perceived discrimination and psychosis: a systematic review of the literature. *Social psychiatry and psychiatric epidemiology,**54*, 1023–1044. 10.1007/s00127-019-01729-3 31236631 10.1007/s00127-019-01729-3

[CR25] Ronald, A., Sieradzka, D., Cardno, A. G., Haworth, C. M., McGuire, P., & Freeman, D. (2014). Characterization of psychotic experiences in adolescence using the specific psychotic experiences questionnaire: findings from a study of 5000 16-year-old twins. *Schizophrenia Bulletin,**40*(4), 868–877. 10.1093/schbul/sbt106 24062593 10.1093/schbul/sbt106 PMC4059437

[CR26] Schlier, B., Lincoln, T. M., Kingston, J. L., Gaudiano, S. H., Morris, B. A., & Ellett, L. (2024). Cross-cultural validation of the revised Green et al., paranoid thoughts scale. *Psychological Medicine*. 10.1017/S0033291724000072 38314511 10.1017/S0033291724000072 PMC11413342

[CR27] Statham, V., Emerson, L. M., & Rowse, G. (2019). A systematic review of self-report measures of paranoia. *Psychological Assessment,**31*(2), 139. 10.1037/pas0000645 30234319 10.1037/pas0000645

[CR28] Stefanis, N. C., Hanssen, M., Smirnis, N. K., Avramopoulos, D. A., Evdokimidis, I. K., Stefanis, C. N., & Van Os, J. (2002). Evidence that three dimensions of psychosis have a distribution in the general population. *Psychological Medicine,**32*(2), 347–358. 10.1017/s0033291701005141 11866327 10.1017/s0033291701005141

[CR29] Tennant, R., Hiller, L., Fishwick, R., Platt, S., Joseph, S., Weich, S., Parkinson, J., Secker, S., & Stewart-Brown, S. (2007). The Warwick-Edinburgh Mental Well-being Scale (WEMWBS): development and UK validation. *Health & Quality of Life Outcomes, 5*(63). 10.1186/1477-7525-5-63 10.1186/1477-7525-5-63 PMC2222612 18042300

[CR30] Williams, D. R., Yu, Y., Jackson, J. S., & Anderson, N. B. (1997). Racial differences in physical and mental health: Socio-economic status, stress and discrimination. *Journal of Health Psychology,**2*(3), 335–351. 10.1177/135910539700200305 22013026 10.1177/135910539700200305

[CR31] Williams, T. F., Walker, E. F., Strauss, G. P., Woods, S. W., Powers, A. R., Corlett, P. R., & Mittal, V. A. (2023). The reliability and validity of the revised Green et al. paranoid thoughts scale in individuals at clinical high-risk for psychosis. *Acta Psychiatrica Scandinavica,**147*(6), 623–633. 36905387 10.1111/acps.13545 PMC10463775

[CR32] Wong, K. K., Freeman, D., & Hughes, C. (2014). Suspicious young minds: paranoia and mistrust in 8-to 14-year-olds in the UK and Hong Kong. *The British Journal of Psychiatry,**205*(3), 221–229. 10.1192/bjp.bp.113.135467 25012682 10.1192/bjp.bp.113.135467

[CR33] Woods, S. W., Walsh, B. C., Addington, J., Cadenhead, K. S., Cannon, T. D., Cornblatt, B. A., & McGlashan, T. H. (2014). Current status specifiers for patients at clinical high risk for psychosis. *Schizophrenia Research,**158*(1–3), 69–75. 10.1016/j.schres.2014.06.022 25012147 10.1016/j.schres.2014.06.022 PMC4152558

