# Supplementary material for: Measuring Paranoid Beliefs in Adolescents: A Comparison of the Revised-Green et al.’s Paranoid Thoughts Scale (R-GPTS) and the Bird Checklist of Adolescent Paranoia (B-CAP)
Source: Res Child Adolesc Psychopathol. 2024 Apr 3;52(8):1319–27. doi: 10.1007/s10802-024-01187-9 (PMC11289251; doi:10.1007/s10802-024-01187-9)
Supplement: Supplementary file 1 — Supplementary Material 1 [file 10802_2024_1187_MOESM1_ESM.docx]

**Table S1.**

**Descriptive values for all self-report questionnaires and scale-reliability in the current sample.**

|  | Descriptive values | | | | | | Reliability | |
| --- | --- | --- | --- | --- | --- | --- | --- | --- |
| Scale/Subscale | Mean | SD | Range | theoretical Range | Skew | Kurtosis | Cronbachs α | ω_h_ |
| Paranoia Measures |  |  |  |  |  |  |  |  |
| RGPTS Ideas of reference | 7.14 | 8.63 | 0 – 32 | 0 – 32 | 1.14 | 0.11 | 0.94 | 0.84 |
| RGPTS Persecutory beliefs | 6.29 | 9.52 | 0 – 38 | 0 – 40 | 1.60 | 1.53 | 0.96 | 0.85 |
| B-CAP Total score | 8.92 | 14.55 | 0 – 89 | 0 – 90 | 2.37 | 6.37 | 0.96 | 0.79 |
| B-CAP Social Harm | 4.40 | 6.62 | 0 – 34 | 0 – 40 | 1.76 | 2.61 | 0.95 | 0.89 |
| B-CAP Conspiracy beliefs | 1.71 | 3.88 | 0 – 25 | 0 – 25 | 3.25 | 11.82 | 0.92 | 0.83 |
| B-CAP Physical Threat | 2.13 | 4.43 | 0 - 25 | 0 – 25 | 2.63 | 7.10 | 0.90 | 0.66 |
| Variables for discriminant validity |  |  |  |  |  |  |  |  |
| DASS Total | 9.41 | 11.99 | 0 – 60 | 0 – 63 | 1.66 | 2.43 | 0.97 | 0.84 |
| DASS Depression | 3.05 | 4.41 | 0 – 21 | 0 – 21 | 1.78 | 2.85 | 0.93 | 0.78 |
| DASS Anxiety | 2.47 | 3.75 | 0 – 20 | 0 – 21 | 1.88 | 3.12 | 0.89 | 0.81 |
| DASS Stress | 3.89 | 4.46 | 0 – 21 | 0 – 21 | 1.30 | 1.18 | 0.91 | 0.73 |
| WEMWBS Wellbeing | 36.24 | 12.15 | 0 – 56 | 0 – 56 | -0.60 | 0.35 | 0.96 | 0.86 |
| Bullying | 2.09 | 3.53 | 0 – 24 | 0 – 25 | 2.92 | 10.24 | 0.88 | 0.72 |
| Discrimination experiences | 5.67 | 7.06 | 0 – 25 | 0 – 30 | 1.40 | 1.08 | 0.92 | 0.82 |

**Table S2.**

**Item statistics for the R-GPTS items**

|  |  | | | Distribution responses by answer option (in %) | | | | |
| --- | --- | --- | --- | --- | --- | --- | --- | --- |
| # | Item (content abbreviated) | Mean | SD | 0  not at all | 1 | 2 | 3 | 4  totally |
|  | Ideas of reference subscale |  |  |  |  |  |  |  |
| 1 | Thinking about friends gossiping about me. | 0.90 | 1.26 | 57.7% | 15.0% | 13.7% | 6.9% | 6.7% |
| 2 | Heard people referring to me. | 0.88 | 1.24 | 58.6% | 15.6% | 9.3% | 12.4% | 4.1% |
| 3 | Upset by friends/colleagues judging me. | 0.87 | 1.25 | 60.5% | 11.5% | 13.0% | 10.% | 5.0% |
| 4 | People laughed at me behind my back. | 0.87 | 1.28 | 59.9% | 15.4% | 9.3% | 8.5% | 6.9% |
| 5 | Thinking about people avoiding me. | 0.70 | 1.20 | 68.1% | 12.1% | 5.9% | 9.3% | 4.6% |
| 6 | People have been dropping hints for me. | 0.56 | 1.04 | 71.6% | 11.9% | 8.7% | 4.6% | 3.3% |
| 7 | Certain people were not what they seemed. | 1.28 | 1.49 | 49.0% | 11.7% | 15.6% | 10.0% | 13.7% |
| 8 | People talking behind my back upset me. | 1.08 | 1.44 | 56.0% | 11.9% | 11.1% | 10.4% | 10.6% |
|  | Persecutory Beliefs subscale |  |  |  |  |  |  |  |
| 9 | Certain individuals have had it in for me. | 0.88 | 1.31 | 61.% | 11.9% | 10.2% | 8.9% | 7.2% |
| 10 | People stared at me to feel threatened. | 0.68 | 1.15 | 67.5% | 11.9% | 9.5% | 6.9% | 4.1% |
| 11 | People did things in order to annoy me. | 1.00 | 1.32 | 55.7% | 12.6% | 14.1% | 11.1% | 6.5% |
| 12 | There was a conspiracy against me. | 0.48 | 1.01 | 76.4% | 9.8% | 6.1% | 5.0% | 2.8% |
| 13 | I was sure someone wanted to hurt me. | 0.53 | 1.10 | 77.0% | 7.4% | 5.6% | 5.9% | 4.1% |
| 14 | People wanted to confuse me. | 0.42 | 0.94 | 78.7% | 9.8% | 5.2% | 3.7% | 2.6% |
| 15 | I was distressed by being persecuted. | 0.41 | 0.88 | 77.4% | 11.3% | 5.6% | 4.3% | 1.3% |
| 16 | People wanted to make me feel bad. | 0.59 | 1.09 | 71.4% | 12.1% | 6.7% | 6.1% | 3.7% |
| 17 | People have been hostile towards. | 0.72 | 1.20 | 66.6% | 11.9% | 9.3% | 6.9% | 5.2% |
| 18 | Angry that someone wanted to hurt me. | 0.59 | 1.13 | 72.9% | 9.8% | 6.9% | 6.1% | 4.3% |

**Table S3.**

**Item statistics for the B-CAP items**

|  |  | | | Distribution responses by answer option (in %) | | | | |  |
| --- | --- | --- | --- | --- | --- | --- | --- | --- | --- |
| # | Item (content abbreviated) | Mean | SD | 0  never | 1  once | 2  couple of times | 3  few times a week | 4  every day | 5  all the time |
|  | Social harm subscale |  |  |  |  |  |  |  |  |
| 1 | People at school make me feel unwanted. | 0.69 | 1.199 | 68.1% | 10.4% | 13.2% | 3.5% | 2.6% | 2.2% |
| 2 | People are gossip about me on social media. | 0.68 | 1.184 | 68.1% | 11.5% | 11.3% | 4.3% | 3.3% | 1.5% |
| 3 | Being pushed out of conversations on purpose. | 0.59 | 1.058 | 69.8% | 11.7% | 10.8% | 4.8% | 2.4% | 0.4% |
| 4 | Friends/partner ignore my messages. | 0.51 | 0.993 | 73.8% | 10.2% | 11.1% | 2.6% | 1.5% | 0.9% |
| 5 | People are trying to embarrass me in class. | 0.57 | 1.076 | 72.2% | 9.8% | 10.2% | 5.0% | 2.0% | 0.9% |
| 6 | People make sly comments. | 0.68 | 1.106 | 65.9% | 12.4% | 12.8% | 6.5% | 1.5% | 0.9% |
| 7 | People lie to me on purpose. | 0.69 | 1.131 | 64.9% | 13.2% | 15.4% | 3.0% | 1.5% | 2.0% |
| 8 | People say things under their breath. | 0.69 | 1.103 | 65.9% | 10.6% | 15.8% | 5.0% | 1.7% | 0.9% |
|  | Conspiracy subscale |  |  |  |  |  |  |  |  |
| 9 | Nasty tricks are played on me. | 0.36 | 0.831 | 79.4% | 11.1% | 5.6% | 2.6% | 0.9% | 0.4% |
| 10 | People try to confuse me. | 0.42 | 0.937 | 77.9% | 10.4% | 6.9% | 2.2% | 2.0% | 0.7% |
| 11 | Groups plan against me. | 0.35 | 0.945 | 83.5% | 6.7% | 5.4% | 1.7% | 0.9% | 1.7% |
| 12 | People collect information or photos against me. | 0.28 | 0.858 | 86.8% | 6.1% | 3.3% | 1.7% | 0.9% | 1.3% |
| 13 | People seek revenge on me. | 0.30 | 0.894 | 85.2% | 6.9% | 4.1% | 1.3% | 0.7% | 1.7% |
|  | Physical harm subscale |  |  |  |  |  |  |  |  |
| 14 | Feeling like being followed/stalked. | 0.26 | 0.859 | 88.7% | 3.7% | 3.5% | 1.5% | 1.7% | 0.9% |
| 15 | Scared of what strangers will do. | 0.53 | 1.152 | 76.8% | 7.4% | 8.5% | 2.8% | 2.2% | 2.4% |
| 16 | People will try to kidnap me. | 0.29 | 0.879 | 87.0% | 4.1% | 5.0% | 1.5% | 1.3% | 1.1% |
| 17 | I could be attacked at any time. | 0.51 | 1.141 | 77.7% | 7.4% | 7.8% | 2.6% | 2.2% | 2.4% |
| 18 | Feeling unsafe around people. | 0.52 | 1.141 | 77.4% | 7.8% | 6.3% | 4.1% | 2.4% | 2.0% |
